# Study of spin-ordering and spin-reorientation transitions in hexagonal manganites through Raman spectroscopy

**DOI:** 10.1038/srep13366

**Published:** 2015-08-24

**Authors:** Xiang-Bai Chen, Nguyen Thi Minh Hien, Kiok Han, Ji-Yeon Nam, Nguyen Thi Huyen, Seong-Il Shin, Xueyun Wang, S. W. Cheong, D. Lee, T. W. Noh, N. H. Sung, B. K. Cho, In-Sang Yang

**Affiliations:** 1 School of Science and Laboratory of Optical Information Technology, Wuhan Institute of Technology, Wuhan 430205, China; 2 Department of Nano Science & Mechanical Engineering and Nanotechnology Research Center, Konkuk University, Chungju 380-701, Korea; 3 Department of Physics and Division of Nano-Sciences, Ewha Womans University, Seoul 120-750, Korea; 4 Rutgers Center for Emergent Materials and Department of Physics & Astronomy, Rutgers University, Piscataway, New Jersey 08854, USA; 5 Center for Correlated Electron Systems, Institute for Basic Science (IBS), Seoul 151-742, Republic of Korea; 6 Department of Physics and Astronomy, Seoul National University (SNU), Seoul 151-742, Republic of Korea; 7 School of Materials Science and Engineering, Gwangju Institute of Science and Technology (GIST), Gwangju 500-712, Korea; 8 Department of Photonics and Applied Physics, Gwangju Institute of Science and Technology (GIST), Gwangju 500-712, Korea

## Abstract

Spin-wave (magnon) scattering, when clearly observed by Raman spectroscopy, can be simple and powerful for studying magnetic phase transitions. In this paper, we present how to observe magnon scattering clearly by Raman spectroscopy, then apply the Raman method to study spin-ordering and spin-reorientation transitions of hexagonal manganite single crystal and thin films and compare directly with the results of magnetization measurements. Our results show that by choosing strong resonance condition and appropriate polarization configuration, magnon scattering can be clearly observed, and the temperature dependence of magnon scattering can be simple and powerful quantity for investigating spin-ordering as well as spin-reorientation transitions. Especially, the Raman method would be very helpful for investigating the weak spin-reorientation transitions by selectively probing the magnons in the Mn^3+^ sublattices, while leaving out the strong effects of paramagnetic moments of the rare earth ions.

Phase transition is a subject of great interest in physics. Raman spectroscopy of phonon scattering has been widely applied to investigate crystallographic phase transitions^1^,^2^,^3^,^4^,^5^; it offers an opportunity as a sensitive probe for the study of crystal structure changes. In magnetic materials, Raman spectroscopy of phonon scattering can also be applied to investigate magnetic phase transitions if spin-phonon coupling has clear influence on phonon scattering^6^,^7^,^8^,^9^,^10^,^11^. Generally spin-phonon coupling has weak influence on phonon scattering; thus Raman study of phonons could only be helpful to investigate spin-ordering transitions. Raman spectroscopy of magnon scattering, when observed, can directly investigate the spin properties, thus would be powerful to study not only spin-ordering transitions, but also weaker spin-reorientation transitions. However, in general, magnon scattering has much weaker intensity comparing with phonon scattering, which makes it difficult to apply Raman study of magnon scattering to investigate spin-ordering and spin-reorientation transitions^2^,^12^. In strong resonance condition, magnon scattering can be significantly enhanced. Then by selecting appropriate polarization configuration to reduce strong phonon scattering signal, magnon scattering can be clearly observed. Strong signal of magnon scattering would be very helpful for studying magnetic phase transitions by Raman spectroscopy.

The most direct method for studying spin-ordering and spin-reorientation transitions in magnetic materials would be through magnetization measurements. In practice, magnetization measurements are subject to several restrictions. Magnetization method is difficult to study thin film samples, since the magnetization contribution due to the substrate would be very difficult to be subtracted. Also magnetization method is difficult to study spin-reorientation transitions, since spin-reorientation would be dependent on the applied magnetic field. Inelastic neutron scattering can provide information on magnetic excitations, is another powerful technique that can directly investigate spin-ordering and spin-reorientation transitions. However, inelastic neutron scattering measurements have been restricted to rather thick samples^13^,^14^. Elastic neutron scattering such as neutron diffraction and neutron reflectivity measurements are also very useful to study magnetic phase transitions^15^,^16^,^17^. Neutron reflectivity experiments can be applied to very thin samples, but magnetic origin reflection would be weak and not well resolved, thus very difficult to observe spin-reorientation transitions^17^. Neutron diffraction had been the most powerful experimental tool to study both spin-ordering and spin-reorientation transitions, but large samples need to be used^16^. It was demonstrated that optical second harmonic generation spectroscopy is an important supplement to neutron diffraction for studying both spin-ordering and spin-reorientation transitions, while also large samples need to be used^18^. In this paper, we show that, when magnon scattering is clearly observed, temperature dependent Raman study of magnon scattering provides a simple and powerful method for investigating both spin-ordering and spin-reorientation transitions in single crystal as well as thin film samples.

## Results

**Figure 1:**
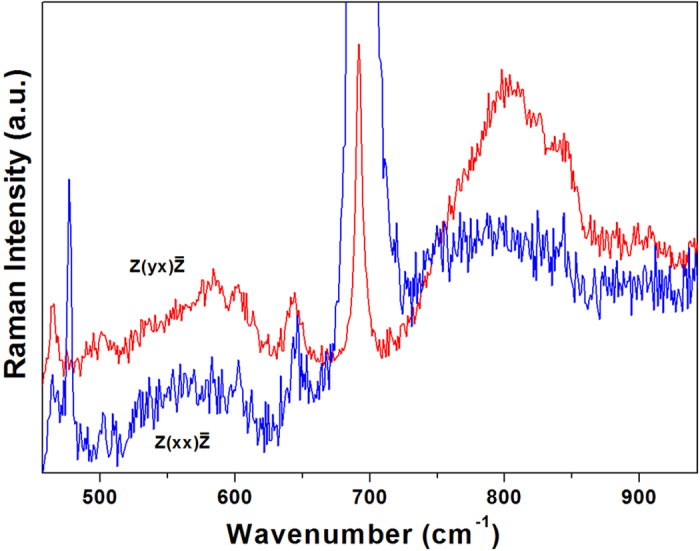
Polarized Raman spectra of hexagonal LuMnO_3_ single crystal at 21 K obtained in the 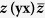 and 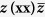 configurations using 633 nm red laser. The broad band of 710–880 cm^−1^ in single crystal is much more asymmetric than that in thin film. This may indicate more possible modes of magnon scattering with similar energies in single crystal. Mn^3+^ has S = 2, “multi-spin-flipping” magnon mode could be excited in one or more Mn^3+^ ions.

Figure 1 shows the polarized Raman scattering spectra of hexagonal LuMnO_3_ single crystal obtained at 21 K in the 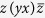 and 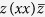 configurations with the excitation source of 633 nm laser. The strong narrow peaks at 693 cm^−1^ and 476 cm^−1^ can be assigned to A_1_ phonon modes; the weak narrow peaks at 645 cm^−1^, 500 cm^−1^, and 465 cm^−1^ can be assigned to E_2_ phonon modes^19^,^20^. The broad bands of 520–630 cm^−1^ and 710–880 cm^−1^ would be mainly originated from magnon scattering^21^,^22^,^23^,^24^,^25^. The Raman spectroscopy of hexagonal manganites has been extensively studied, these studies were focused on phonon vibrations and spin-phonon coupling^6^,^7^,^8^,^19^,^20^,^26^,^27^,^28^. The Raman studies of magnon scattering in hexagonal manganites were attempted more than a decade ago^29^,^30^. Until recently, the observation of magnon scattering was identified by our studies of hexagonal manganite thin films^21^,^22^,^23^,^24^,^25^. The observation in single crystal further confirms the intrinsic magnon scattering origin of the broad bands in Fig. 1, but not due to surface, interface, or defect-related effects which may be possible in thin film samples.

As can be seen in Fig. 1, the magnon scattering can be much more easily observed in 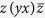 configuration than in 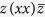 configuration. This would be correlated with the following two factors. First, the very strong A_1_ phonon at 693 cm^−1^ in 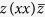 configuration is significantly reduced by selection rule in 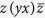 configuration. Second, the Raman cross section of the observed magnon scattering would be larger in 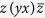 configuration than that in 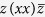 configuration. Similar to our results, in cuprite superconductors, it was reported that the Raman cross section of two-magnon scattering is significantly larger in 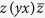 configuration than that in 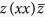 configuration^31^. In antiferromagnetic materials, even-order magnon scattering is typically observed^32^. Using Heisenberg Hamiltonian model, we have estimated that the broad band of 520–630 cm^−1^ would be mainly correlated with 2-spin-flipping magnon scattering, and the broad band of 710–880 cm^−1^ would be mainly correlated with 4-spin-flipping magnon scattering. The explanation of large Raman cross section of 4-spin-flipping magnon scattering would need further theoretical study. In this paper, we simply apply the experimental observation of magnon scattering to investigate magnetic phase transitions.

**Figure 2:**
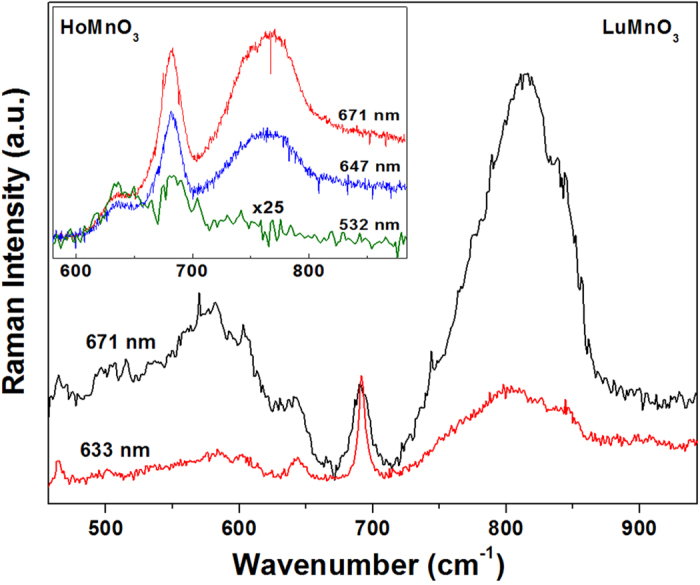
Raman spectra of hexagonal LuMnO_3_ single crystal at 21 K obtained in the 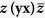 configuration using 671 nm and 633 nm lasers. The intensities are normalized by the intensity of A_1_ phonon at 693 cm^−1^. (The spectra of 671 and 633 nm laser exciations were taken with J-Y T64000 and LabRaman system, respectively. The difference of linewidth between these two spectra would be due to intrinsic linewith broadening by the two different Raman systems.) Inset shows Raman spectra of HoMnO_3_ thin film obtained in the 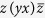 configuration using 671 nm, 647 nm, and 532 nm lasers, taken from our previous work^22^.

The difficulty of observing magnon scattering in hexagonal manganite would be mainly due to narrow energy range of the resonance effect. Figure 2 shows the resonance effect of magnon scattering of hexagonal LuMnO_3_ single crystal with excitations of 671 nm and 633 nm lasers. More systematic resonance effect of magnon scattering was investigated in our previous study of hexagonal HoMnO_3_ thin film (inset of Fig. 2)^22^. Our previous study suggested that the resonance effect of magnon scattering would be strongly correlated with “on-site Coulomb energy”^22^. 671 nm (1.85 eV), 647 nm (1.92 eV), and 633 nm (1.96 eV) lasers have energy within the “on-site Coulomb energy” of hexagonal manganite (Mn d-d transition: ~1.8 eV, linewidth: ~0.15 eV), thus magnon scattering can be observed. While 532 nm (2.33 eV) laser has energy outside the “on-site Coulomb energy”, thus magnon scattering could not be detected. As can be seen in Fig. 2, magnon scattering has much stronger resonance effect than phonon scattering. This suggests that strongest possible resonance effect should be chosen for best observation of magnon scattering, i.e., the laser energy should be close to the peak of “on-site Coulomb energy”.

We have shown that by choosing strong resonance condition and appropriate polarization configuration, magnon scattering can be clearly observed. The clear observation of magnon scattering makes further systematic studies of magnetic properties by Raman spectroscopy possible. Here, we focus on temperature dependent behaviors of magnon scattering for studying spin-ordering and spin-reorientation transitions in hexagonal manganites.

**Figure 3:**
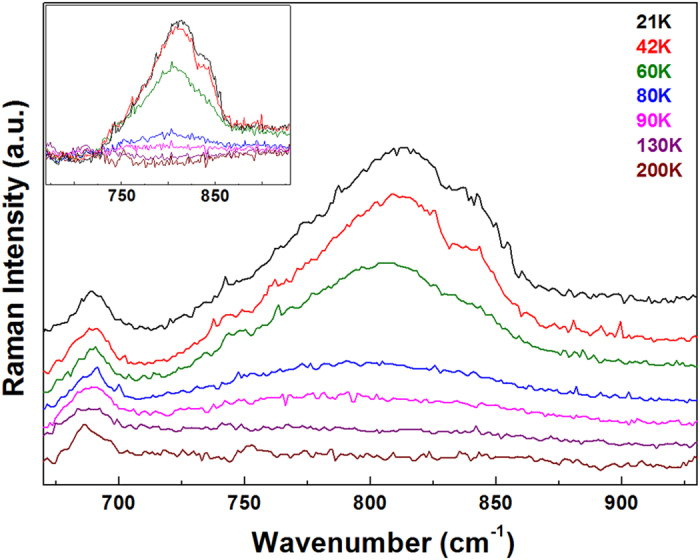
Temperature dependent (20 K–200 K) Raman spectra of hexagonal LuMnO_3_ single crystal in the 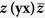 configuration. Only seven representative spectra are shown in the figure. Each spectrum is shifted in y-direction for clarity. The inset shows the difference Raman spectra against the spectrum taken at 110 K.

**Figure 4:**
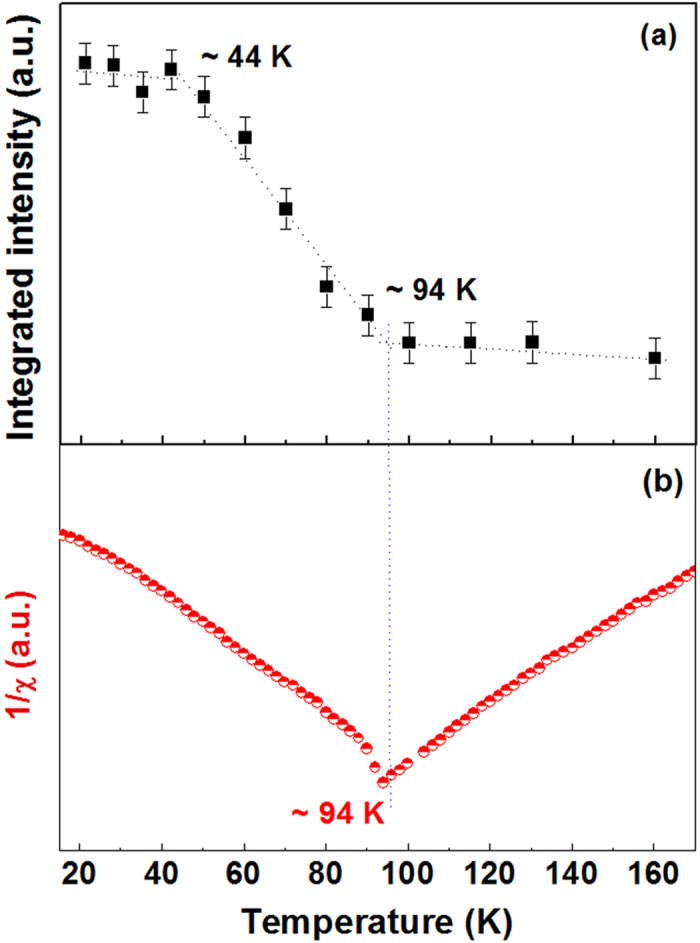
(**a**) Temperature dependence of the integrated intensity of the difference Raman spectra in the range 720–1060 cm^−1^ (filled square), and (**b**) temperature dependence of the magnetization measurement (half-filled circle) of hexagonal LuMnO_3_ single crystal.

The temperature dependence of polarized Raman spectra from hexagonal LuMnO_3_ single crystal obtained in the 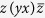 configuration with 671 nm laser is shown in Fig. 3. The magnon scattering intensity is very sensitive to the temperature, thus temperature dependence of magnon scattering intensity would be helpful to deduce spin-ordering and spin-reorientation transition temperatures. Since magnon scattering intensity decreases much more rapidly than that of phonon scattering as temperature increases, using the phonon intensity as a reference would be useful to accurately investigate temperature dependence of magnon scattering. Thus, we plotted the intensity difference spectra, as shown in the inset of Fig. 3. The intensity difference spectra were obtained by, first normalizing all the spectra using the intensity of the A_1_ phonon as a reference, and then taking the difference of the intensity at a particular temperature and that of the spectrum at any temperature above the Néel temperature, for example, 110 K. To observe the temperature dependent behavior of magnon scattering intensity more clearly, we plotted the temperature dependence of the integrated intensity in the range 720–1060 cm^−1^, as presented in Fig. 4(a). Interestingly, Fig. 4(a) indicates two magnetic phase transitions at 94 K and 44 K in hexagonal LuMnO_3_ single crystal.

The magnetic properties of hexagonal manganites *R*MnO_3_ (*R* = rare earth) mainly arise from the Mn^3 + ^(3d^4^) ions with spin *S* = 2. The antiferromagnetic spin-ordering transition in the *a*-*b* plane of the Mn^3 + ^spins occurs at Néel temperature ~70–130 K, depending on the rare earth ion^33^,^34^,^35^. Including the antiparallel and parallel orientation of corresponding Mn^3 + ^spins at *z* = 0 and *z* = *c*/2 in the unit cell, there are a few different possible magnetic symmetries^18^. Below Néel temperature, the geometrical frustration of Mn^3 + ^spins could lead to a different symmetry arrangement of triangular magnetic ordering, i.e., spin-reorientation transition could occur. Such a long-range magnetic interaction depends not only on the temperature and magnetic field, but also factors such as the size of the rare earth ion, interatomic distances, and angles in the structure^36^,^37^. Smaller rare earth ions lead to more than one spin-reorientation transition due to relatively enhanced geometrical frustration^37^. Thus, the spin-reorientation transition would be more easily observed in smaller rare earth ion hexagonal manganites.

To investigate antiferromagnetic spin-ordering transition, magnetization measurements are commonly applied. The magnetization of hexagonal LuMnO_3_ single crystal sample is shown in Fig. 4(b), which clearly indicates T_N_ = 94 K of hexagonal LuMnO_3_. Our Raman result shown in Fig. 4(a) agrees well with the magnetization result in Fig. 4(b). Also, the value of T_N_ = 94 K agrees with the previously reported Néel temperature of hexagonal LuMnO_3_ single crystal^18^. For the spin-reorientation transition, due to its weakness and magnetic field dependence, magnetization measurements would be hard to be employed in the investigation. As can be seen in Fig. 4(b), spin-reorientation transition could not be detected in hexagonal LuMnO_3_ single crystal with applied magnetic field *H* = 1000 Oe. In hexagonal LuMnO_3_ nanocrystals, weak magnetic anomaly due to spin-reorientation transition had been observed at ~44 K. But the anomaly value depends on applied magnetic field, and with high applied magnetic field at 20 kOe anomaly disappeared^37^. Nonlinear optical method (second harmonic generation experiments) had been applied to determine the magnetic symmetry of hexagonal manganites^18^. The nonlinear optical method showed higher spin-reorientation transition temperature of hexagonal LuMnO_3_ single crystal than that of LuMnO_3_ nanocrystals by magnetization measurement.

Figure 4(a) indicates that our Raman method can easily deduce the spin-ordering and spin-reorientation transition temperatures of hexagonal LuMnO_3_ single crystal. The magnon scattering is intrinsic properties of magnetic material, and the intensity of magnon scattering would be very sensitive to the change of magnetic symmetry. Therefore, the Raman method provides a simple and powerful technique to study both spin-ordering and spin-reorientation transitions in magnetic materials.

**Figure 5:**
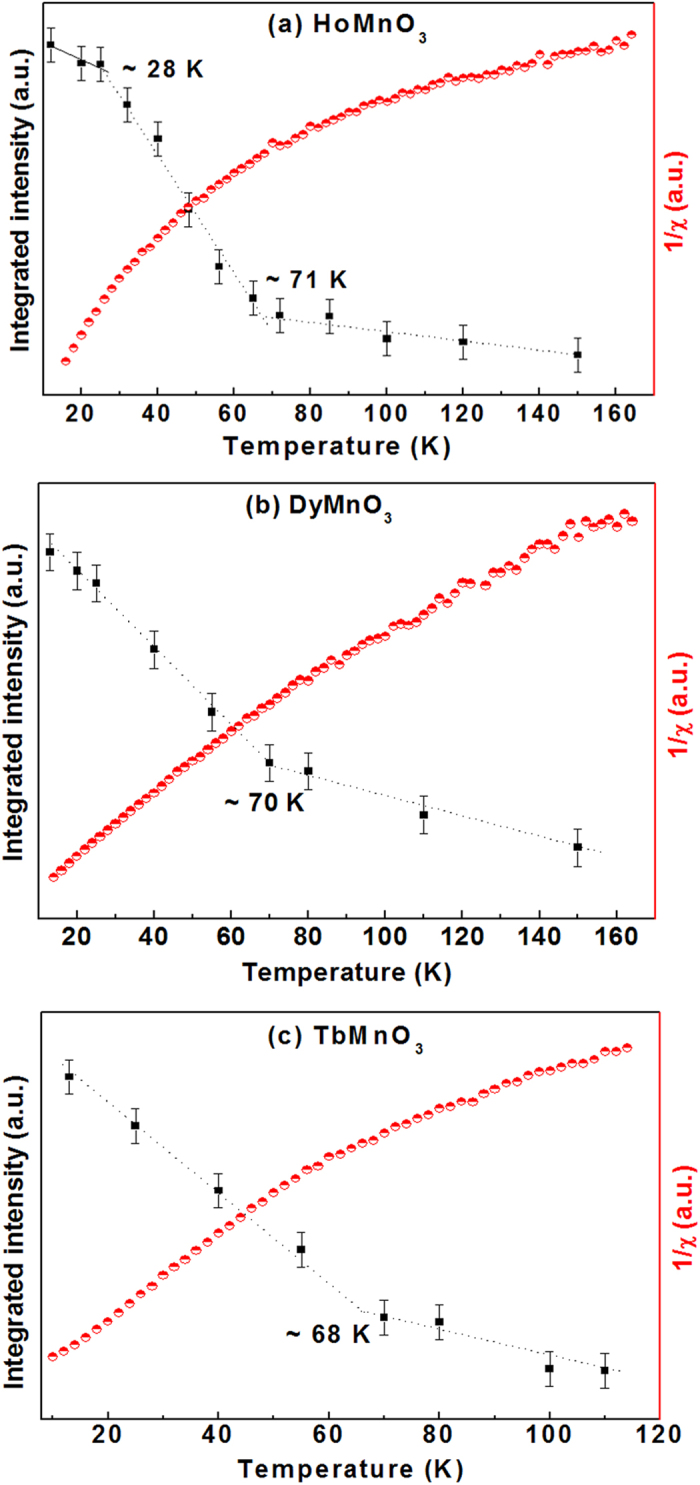
Temperature dependence of the integrated intensity of the difference Raman spectra in the range 700–1000 cm^−1^ (filled square) and of magnetization measurement (half-filled circle) of (**a**) HoMnO_3_, (**b**) DyMnO_3_, and (**c**) TbMnO_3_ thin films.

Above we have discussed the Raman method for investigating spin-ordering and spin-reorientation transitions in single crystal. Now, we apply the Raman method for thin film samples. Figure 5 shows the temperature dependence of integrated magnon scattering intensity of hexagonal HoMnO_3_, DyMnO_3_, and TbMnO_3_ thin films together with the magnetization measurements. For HoMnO_3_ thin film, the Raman method indicates two magnetic phase transitions: an antiferromagnetic spin-ordering transition at 71 K and a spin-reorientation transition at 28 K. These values are smaller than the values reported in HoMnO_3_ single crystal^18^, which would be mainly correlated with the tensile stress in HoMnO_3_ thin film. For DyMnO_3_ and TbMnO_3_ thin films, the Raman method indicates only an antiferromagnetic spin-ordering transition at 70 and 68 K, respectively.

It had been reported that manganites with smaller rare earth ions would show spin reorientation transition below Néel ordering, while manganites with larger rare earth ions would not show any spin reorientation transition^18^,^37^. Our results agree very well with these reports. For LuMnO_3_ and HoMnO_3_ with smaller rare earth ions, both antiferromagnetic spin-ordering and spin-reorientation transitions are observed; while for DyMnO_3_ and TbMnO_3_ with larger rare earth ions, antiferromagnetic spin-ordering transition is observed, spin-reorientation transition is not observed.

For all the HoMnO_3_, DyMnO_3_, and TbMnO_3_ thin film samples, Fig. 5 shows that it is quite difficult to deduce the magnetic phase transition temperatures (even the Néel ordering temperature) from the magnetization measurement. One reason of the difficulty of magnetization measurement is the existence of *f* moments of the rare earth ions in these compounds^38^,^39^. These *f* moments yield a large paramagnetic component in the magnetization and mask the anomaly arising from the ordering of the spins associated with the Mn^3 + ^ions. Another reason lies with the substrate effect on the magnetization of the thin film samples. In the magnetization measurement, both thin film and substrate are probed at the same time, thus the magnetization curve contains both information of thin film and the substrate. However, the magnetization contribution due to substrate would be very difficult to be subtracted, since the interface effect between substrate and thin film would also change the magnetization curve. So, it would be difficult to observe intrinsic magnetic phase transitions in thin films by magnetization measurement.

Figure 5, however, show that the Raman method can clearly show both spin-ordering and spin-reorientation transitions in thin film samples. In Raman measurements, only the surface within the skin-depth of thin film is probed, thus it investigates the intrinsic properties of the thin film. In addition, the magnons in the Mn^3 + ^ sublattices are selectively probed while bypassing the effects of paramagnetic *f*–electron moments of the rare earth ions in these compounds. Therefore, the Raman study of magnon scattering would be very helpful to investigate magnetic phase transitions in hexagonal manganites and, possibly, other magnetic materials.

## Discussion

We have discussed that by choosing strong resonance condition and appropriate polarization configuration, magnon scattering can be clearly observed by Raman spectroscopy, then the temperature dependence study of magnon scattering provides a simple and powerful method for investigating magnetic phase transitions. The advantages of the Raman methods for investigating magnetic phase transitions are: (i) the capability of studying not only spin-ordering transition, but also spin-reorientation transition; (ii) very useful for studying magnetic phase transitions in thin film samples, since it probes within the skin-depth of the thin films, thus provides intrinsic properties of the thin films; (iii) very useful for studying magnetic nanomaterials, since Raman measurement only needs microgram or less of sample.

In addition the advantages of studying magnetic phase transitions, the Raman method would be very powerful for investigating magnetic, crystallographic, and electronic phase transitions simultaneously. This would be very important for understanding complex phase transitions in solids, such as the mystery of phase transition mechanisms in V_2_O_3_, which has been investigated for more than half a century but still not fully understood^12^.

Following our method for investigating magnetic phase transitions in hexagonal manganites, the Raman method can be widely applied for investigating various phase transition problems, especially the magnetic phase transitions in thin films and nanomaterials. The general steps for applying Raman spectroscopy to study magnetic phase transitions are: (1) choose the excitation source close to the peak of “on-site Coulomb energy” of the material to gain strongest resonance effect of magnon scattering; (2) choose the appropriate polarization configuration to achieve larger Raman cross section of magnon scattering and reduce strong phonon scattering signal, thus observe magnon scattering clearly; (3) perform temperature dependent Raman experiments; (4) investigate the temperature dependent behaviors of magnon scattering to study magnetic phase transitions.

## Methods

**Hexagonal LuMnO**_**3**_ **single crystal** was grown using the traveling floating zone method and characterized by magnetization, resistivity, and x-ray powder diffraction. The lattice constants as well as the observed macroscopic properties agree well with measurements reported in literature. Platelet single crystal sample was cleaved perpendicular to the *c* axis.

**Hexagonal RMnO**_**3**_ **(R = Ho, Dy, Tb) thin films** were grown on Pt(111)//Al_2_O_3_ (0001) substrates by pulsed laser deposition techniques. All the thin films were grown expitaxially with their *c* axis perpendicular to the film surface. Note that in the bulk form, DyMnO_3_ and TbMnO_3_ are in orthorhombic phases. The epistabilization technique is used to convert these materials into hexagonal form. The high-resolution transmission electron microscopy (HRTEM) indicated the formation of good quality crystalline structure on the Pt layer with a well defined interface and clear atomic arrangements.

**Polarized Raman spectra** were obtained in backscattering configurations with Jobin Yvon T64000 and LabRaman systems. The T64000 system has a 671 nm excitation laser, and the LabRaman system has a 633 nm excitation laser. The propagation of laser beam is along the *c*-axis of hexagonal manganite samples, i.e., the polarized Raman experiments were performed under 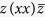 and 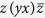 configurations. The samples were mounted in a helium closed cycle cryostat and the sample temperature was varied from 20 to 200 K, and the laser beam power density was low enough to avoid laser heating.

**Temperature-dependent magnetizations** were obtained with a Quantum Design magnetic property measurement system in the temperature range 10–160 K. The sample was zero-field cooled (ZFC) to 10 K. Magnetization measurements, with the field parallel to the *ab* plane, were performed while warming the sample up to 160 K under *H* = 1000 Oe.

## Additional Information

**How to cite this article**: Chen, X.-B. *et al.* Study of spin-ordering and spin-reorientation transitions in hexagonal manganites through Raman spectroscopy. *Sci. Rep.* **5**, 13366; doi: 10.1038/srep13366 (2015).
